# Visualization of Gli Activity in Craniofacial Tissues of Hedgehog-Pathway Reporter Transgenic Zebrafish

**DOI:** 10.1371/journal.pone.0014396

**Published:** 2010-12-21

**Authors:** Tyler Schwend, Evyn J. Loucks, Sara C. Ahlgren

**Affiliations:** 1 Integrated Graduate Program, Northwestern University Feinberg School of Medicine, Chicago, Illinois, United States of America; 2 Department of Pediatrics, Northwestern University Feinberg School of Medicine, Chicago, Illinois, United States of America; 3 Developmental Biology Program, Children's Memorial Research Center, Chicago, Illinois, United States of America; University of Colorado, Boulder, United States of America

## Abstract

**Background:**

The Hedgehog (Hh)-signaling pathway plays a crucial role in the development and maintenance of multiple vertebrate and invertebrate organ systems. Gli transcription factors are regulated by Hh-signaling and act as downstream effectors of the pathway to activate Hh-target genes. Understanding the requirements for Hh-signaling in organisms can be gained by assessing Gli activity in a spatial and temporal fashion.

**Methodology/Principal Findings:**

We have generated a Gli-dependent (Gli-d) transgenic line, Tg(Gli-d:mCherry), that allows for rapid and simple detection of Hh-responding cell populations in both live and fixed zebrafish. This transgenic line expresses a mCherry reporter under the control of a Gli responsive promoter, which can be followed by using fluorescent microscopy and *in situ* hybridization. Expression of the mCherry transgene reporter during embryogenesis and early larval development faithfully replicated known expression domains of Hh-signaling in zebrafish, and abrogating Hh-signaling in transgenic fish resulted in the suppression of reporter expression. Moreover, ectopic *shh* expression in Tg(Glid:mCherry) fish led to increased transgene production. Using this transgenic line we investigated the nature of Hh-pathway response during early craniofacial development and determined that the neural crest skeletal precursors do not directly respond to Hh-signaling prior to 48 hours post fertilization, suggesting that earlier requirements for pathway activation in this population of facial skeleton precursors are indirect.

**Conclusion/Significance:**

We have determined that early Hh-signaling requirements in craniofacial development are indirect. We further demonstrate the Tg(Gli-d:mCherry) fish are a highly useful tool for studying Hh-signaling dependent processes during embryogenesis and larval stages.

## Introduction

Members of the highly conserved Hedeghog (Hh)-family of secreted proteins are essential in a variety of processes in vertebrate and invertebrate development. Hh-ligands function dually as morphogens, to pattern cells in a multicellular target field, as well as mitogens, to control cellular proliferation and survival. Understanding the many orchestrated events that transduce the Hh-signal has been of considerable interest to scientists and clinicians, as perturbations to the Hh-signaling pathway leads to a variety of developmental disorders [1], [2], while unchecked Hh-pathway regulation can develop into a variety of cancers [3], [4].

Hh-signaling is initiated upon Hh-ligand binding to the large, hydrophilic extracellular loops of its cognate twelve-pass transmembrane receptor Patched (Ptch) [5]–[7] to relieve inhibition of a seven-pass transmembrane receptor protein Smoothened (Smo) [8], [9]. Stimulation of cellular Smo leads to signal transduction events involving the activation of the Gli family of transcription factors [10], [11]. Gli proteins are zinc finger transcription factors that respond to Hh-signals by regulating the transcription of Hh-target genes [12]. In zebrafish, four *gli* genes (*gli1*, *gli2a*, *gli2b*, *gli3*) have been identified and implicated in early development [13]–[19]. Hh-target gene expression may become upregulated or downregulated by Glis, as activator and repressor functions are distributed across the four zebrafish proteins, thereby creating a complex code of Hh-dependent gene transcription that influences cell proliferation and differentiation during early development. Hh-dependent gene transcription is similarly regulated among three Gli proteins (Gli1, Gli2, Gli3) in mouse [20]–[27] and the single bifunctional *gli* homolog Cubitus interruptus (*ci*) in *Drosophila* [28], [29]. The dual transcription activator/repressor functions are best understood for *ci*. In the absence of Hh-signals, Ci is cleaved into a truncated repressor form that inhibits expression of Hh-target genes. On the other hand, in the presence of Hh-signals, Ci protein remains as a full-length activator version that promotes expression of Hh-target genes [28]–[30]. In the mouse, both Gli2 and Gli3 can undergo post-translational proteolytic processing events that likely alter these molecules from full-length activators of Hh-signaling, to C-terminally truncated repressor forms [25], [26], [28], [30]–[32]. In contrast to Gli2 and Gli3, mouse Gli1 activity is not regulated post-translationally. Instead, *Gli1* levels are transcriptionally regulated in tissues responding to Hh-signaling [7], [15], [33]–[37]. Transcriptional regulation of *Gli1* appears to be conserved, as studies in mouse, frog and zebrafish have all shown that *Gli1* regulation is sensitive to changes in Hh-signaling [7], [15], [33]–[36]. In zebrafish, *gli1* is the major activator of Hh-signaling [15], [19], although *gli2a/b* and *gli3* have roles in both activating and repressing the Hh signal, depending on the cellular context [15], [17], [18]. Loss of *gli1* by genetic mutation, or reducing embryonic *gli* levels by antisense morpholino oligonucleotides, nearly phenocopies the ventral neural patterning defects seen in *smu/smo* mutants with complete Hh-signaling loss [15], [38], [39]. Genetic studies in the zebrafish demonstrate that combinations of *gli1*, *gli2a/b*, and *gli3* are needed to precisely integrate Hh-signaling during development [13]–[15], [18].

Gli transcription factors transduce the Hh-signal by binding to DNA in a sequence specific manner through the recognition of the DNA motif GACCACCCA, known as the Gli-consensus binding sequence (Gli-CBS) [10], [11]. One widely utilized approach to show changes in Hh-responsiveness within cells has been to use a Gli-dependent (Gli-d) reporter, whereby an engineered promoter element containing eight repeating Gli-CBS drives expression of a luciferase gene [15], [40]. By measuring luciferase activity in reporter construct-expressing cells, it is possible to determine the levels of cellular Gli activity. While the Gli-d luciferase reporter has been invaluable for analyzing potential agonists and antagonists of the Hh-signaling pathway in a variety of cell lines, it has not been feasible to utilize the reporter construct to determine spatial or temporal locations of Hh-target regions during animal development. As mentioned above, in the zebrafish *gli1* is the major activator of Hh-signaling, and zebrafish Gli1 has been shown to activate the luciferase form of this reporter *in vitro* [15]. In the embryo, the presence of repressor Gli proteins refines Hh-pathway activity by suppressing Gli1-mediated transcriptional activation. In this regard, a positive readout of Hh-signaling is found when Gli activation exceeds Gli repression. Therefore, probing Gli-dependent reporter activity *in vivo* provides a direct reflection of Hh-activity through the binding of Gli-CBS. With this in mind, we reasoned that if the Gli-d reporter construct was engineered to express a fluorescent molecule, instead of luciferase, it would be possible to visualize Gli activity within live, transparent zebrafish embryos. We further reasoned that by creating transgenic zebrafish lines that stably express this construct, we could observe dynamic changes in Hh-signaling within specific organs during normal embryonic and larval development by monitoring reporter transgene expression.

Previous studies, including one recently published by our group, have revealed that Hh-signaling has a variety of separate functions during craniofacial skeleton development in the zebrafish [39], [41]–[43]. Hh-signaling is known to play a prominent role in facial growth and patterning, as evidenced by a significant disease burden within the human face upon pathway attenuation during development. Most notable among these is holoprosencephaly, wherein forebrain deficits are often accompanied by severe craniofacial defects including cyclopia and cleft palate [44], [45]. Loss of function mutations in mouse and zebrafish *Smo* severely reduce jaw outgrowth and lead to the loss of nearly all craniofacial skeleton elements [39], [41], [46]. Genetic mutants with lesser disruptions, however, such as *chameleon* mutant zebrafish with a disrupted *dispatched1* gene have shown distinct requirements for Hh-signaling in cartilage differentiation within the posterior arches [42], [43].

The main Hh-ligand, *sonic hedgehog* (*shh*), is expressed in a variety of craniofacial tissues throughout stages of facial growth and patterning, including the embryonic mesendoderm, and later in the ventral brain, oral ectoderm and pharyngeal endoderm [42], [47]. Each of these tissues provides potential sources of the Hh-ligand that may influence craniofacial development. Zebrafish genetic mosaic studies have revealed requirements for *shh* or its receptor, *smo*, in the oral ectoderm and brain, while temporal pharmacological inhibition studies have helped to define when the Hh-signal is required during development. We recently found that the Hh-pathway must be active during two separate stages of development for normal pharyngeal arch (PA) skeletogenesis. Activity is required first during gastrulation (4–10 hours post fertilization (hpf)), and then again during late pharyngula stage (32–48 hpf), after the cranial neural crest (CNC) cells, which comprise the majority of the head skeletal elements, have migrated into the jaw and PA [42].

The widespread, and dynamic, expression of Hh-ligands in the head, as well as the spectrum of phenotypes that exist depending on both the tissue disruption or the timing of the Hh-signaling deficit, suggest that the Hh-signaling mechanism that controls craniofacial development is complex and multifaceted. Although genetic mutant analysis and temporal inhibition studies have suggested roles for Hh-signaling in facial and neural tissues, these studies have provided limited details concerning when specific tissue groups in the craniofacial region are receiving/responding to Hh-signals. Thus, to better understand the spatial and temporal targets of Hh-signaling in craniofacial development, we have created an *in vivo* reporter system for detecting Gli1 responding cells throughout development. By utilizing the previously described Gli-d reporter construct from Sasaki et al., 1997 that uses eight repeating Gli-CBS to drive expression of a mCherry reporter we have generated a novel transgenic line that can be used to dynamically visualize Hh-responding cells in zebrafish [40]. In this study we have characterized the expression of the Gli-d reporter during early zebrafish craniofacial development. Next, we determined precisely when and where Hh-signals are required during craniofacial development. Further, by performing timed inhibition studies on Tg(Gli-d:mCherry) fish, we determined that Hh-signaling controls cartilage differentiation in CNC precursors residing in the ventral arches by an indirect mechanism. Importantly, this study reveals that we now have a new and powerful tool for determining Hh-signaling targets *in vivo* during development.

## Results

### A Gli-dependent reporter is capable of detecting changes to the Hh-signaling pathway in fish

**Figure 1:**
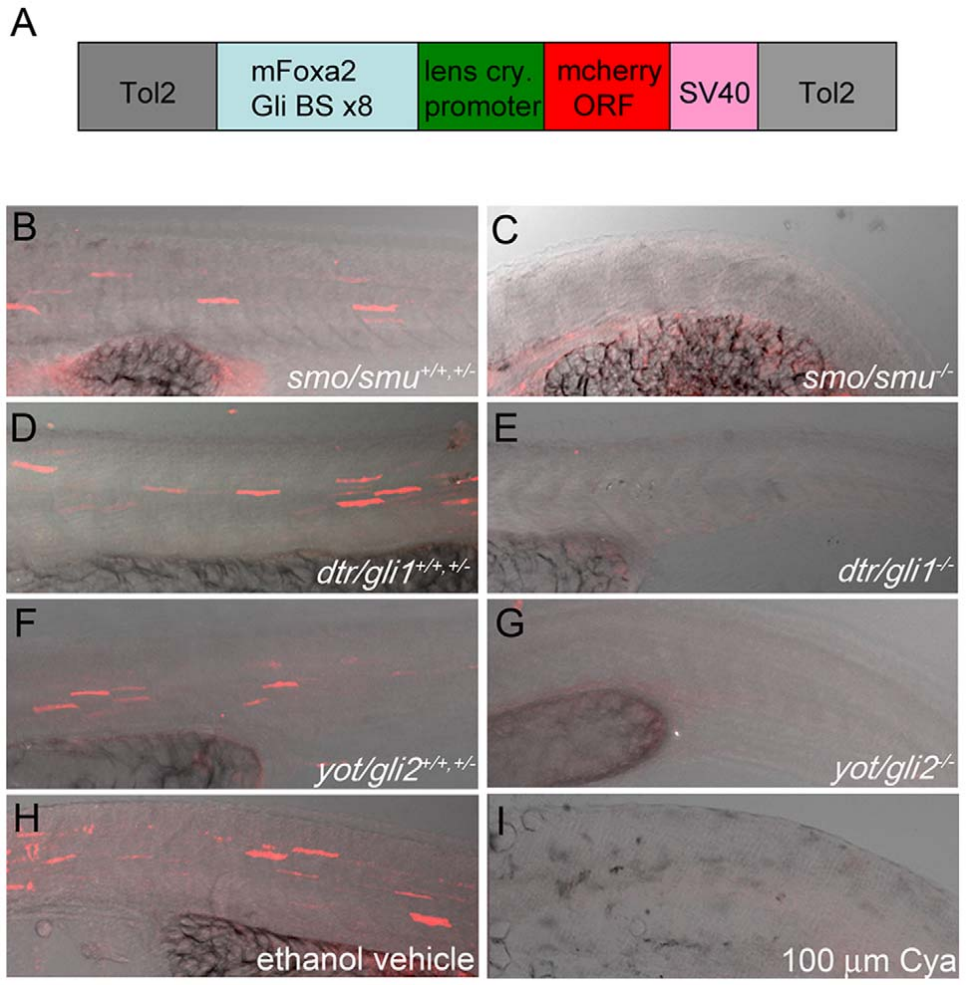
Gli-dependent reporter is capable of detecting modulations to the Hh-signaling pathway when expressed transiently in fish. (A) Schematic of the Gli-d mcherry reporter construct designed in this study. The construct consists of a 0.7 kb fragment encoding eight repeating Gli-CBS from the mouse FoxA2 floor plate enhancer and a minimal lens crystallin promoter (light blue and green respectively) inserted upstream of the mCherry ORF (red). The SV40 polyA signal (pink), which contains a transcriptional termination element, is directly downstream of the mCherry ORF and the entire element is flanked by Tol2 transposable elements (gray). (B–I) Lateral views, anterior to the left, of confocal stack projections of fixed trunk tissue. (B–G) Homozygous genetic mutants (−/−) for *smu/smo* (B,C), *dtr/gli1* (D,E), *yot/gli2* (F,G) were sorted from heterozygotes (+/−) and homozygous wild type (+/+) siblings based on evident characteristic Hh-signaling mutant phenotypes. Genetic mutants (−/−) for Hh-signaling pathway components failed to express mCherry protein (C,E,G), while strong mCherry expression was evident in trunk tissue of (+/+,+/−) siblings (B,D,F). (H,I) Hh-pathway attenuation with 100 µm Cya fully reduced mCherry protein expression in construct-expressing embryos (I) compared to vehicle-treated controls (H).

A Hh-pathway sensitive reporter construct, comprised of a Gli-d promoter element containing eight repeating Gli-CBS from the mouse FoxA2 floor plate enhancer and a minimal lens crystallin promoter, was first shown to faithfully drive luciferase reporter expression in cells activated by Hh-signaling by Sasaki et al., 1997 [40]. Since Gli activity is widely regarded as an indicator of Hh-signaling, we reasoned that a fluorescent mCherry molecule under the control of the Gli-d promoter element would allow live visualization of Hh-pathway activity in live transparent zebrafish embryos, as well as providing a unique marker for Hh-pathway activation in fixed embryos analyzed by both fluorescent imaging and in situ hybridization. A 765 bp fragment of the Gli-d promoter element was PCR amplified, and subsequently cloned into a Gateway 5′ entry vector and used in the Tol2 system for creating transgenic zebrafish [48]. The Gli-d promoter was cloned upstream of the mCherry open reading frame (ORF) and flanked with Tol2 transposable elements (Figure 1A).

To show that the reporter construct could be utilized to identify changes in Hh-signaling pathway activity, we created mosaic transgenic zebrafish by co-injecting 50 pg of Gli-d reporter plasmid DNA and 25 pg of Tol2 mRNA into the one-cell staged progeny of genetic mutant strains deficient in a variety of Hh-signaling components. Following the injections, the embryos were allowed to develop to 30 hpf for analysis. At this developmental stage, homozygous mutants (−/−) for Hh-signaling pathway components could be identified by the presence of U-shaped somites and a prominent ventral tail curl (i.e. Figure 1C). Heterozygotes (+/−) and homozygous wild type (+/+) siblings displayed chevron shaped somites with a straight tail (i.e. Figure 1B). These different phenotypes allowed the embryos to be sorted into mutant (−/−) or wild type (+/+,+/−) categories prior to fixation and imaging. Upon analysis, mCherry fluorescent signals were consistently visible in the ventral brain (data not shown) and within the developing somites of heterozygotes and homozygous wild-type siblings (Figure 1B,D,F). On the other hand, Gli-d reporter expression was undetectable in the homozygous mutant siblings within three separate Hh-signaling mutant lines; *slow-muscle-omitted* (*smu*) mutants, which harbor a mutation in the *smo* receptor (Figure 1C, [38], [39]) the *detour* (*dtr*) mutant which contains a *gli1* loss-of-functon mutation (Figure 1E; [15]) and the *you-too* (*yot*) mutant, encoding a C-terminally truncated Gli2 protein that acts as a dominant repressor of Hh-signaling and is able to block Gli1-mediated transcriptional activation (Figure 1G; [14], [15]). This data strongly suggests that mutant embryos deficient in Hh-signaling fail to express the Gli-d transgene.

The Hh-signaling pathway can be targeted by small molecule chemical compounds. Thus, we next sought to show that the expression of the Gli-d reporter could also be perturbed with a known small molecule inhibitor of the Hh-pathway. Cyclopamine (Cya) acts on the Hh-receptor Smo [49]–[51], upstream of Gli protein activation and nuclear entry, thus making it an ideal pharmacological candidate for testing the Gli-d reporter's potential to reflect Hh-signaling changes. Utilizing a similar strategy for visualizing reporter expression in developing trunk muscle, we saw no levels of reporter expression in wild-type embryos that had been injected as above and subsequently treated with Cya (Figure 1I). In contrast, injected siblings treated with a vehicle control (ethanol) showed robust levels of reporter expression (Figure 1H). In full, these studies clearly show that the Gli-d reporter construct, when applied to fish by transient transfection, can be used to rapidly and reliably detect changes to the Hh-signaling pathway. Using a vertebrate animal model to explore potential modulators of the Hh-signaling pathway, either genetic interactors or small molecules compounds, provides an alternative to performing the same studies *in vitro* in cell lines.

### Generation of Gli-dependent Reporter Transgenic Zebrafish

The fact that the Gli-d reporter was sensitive to Hh-signaling when transiently expressed in fish told us that if the reporter was stably expressed it would be possible to visualize Gli activity dynamically. To generate stable transgenic lines, the construct was co-injected with *in vitro* transcribed Tol2 transposase mRNA [48] into one cell stage wild type embryos. The injected fish were examined for the expected mosaic expression of the transgene at 24 hpf. We identified embryos showing the strongest fluorophore expression within known regions of Hh-signaling activity, such as the forebrain, notochord and muscle fibers (Figure S1). These embryos were used to begin four independent Tg(Gli-d:mCherry) lines. Each independent line displayed identical expression patterns in the craniofacial region throughout embryonic development and during larval stages (data not shown). In-crossing of the novel lines did not reveal any gross transgene-induced defects.

**Figure 2:**
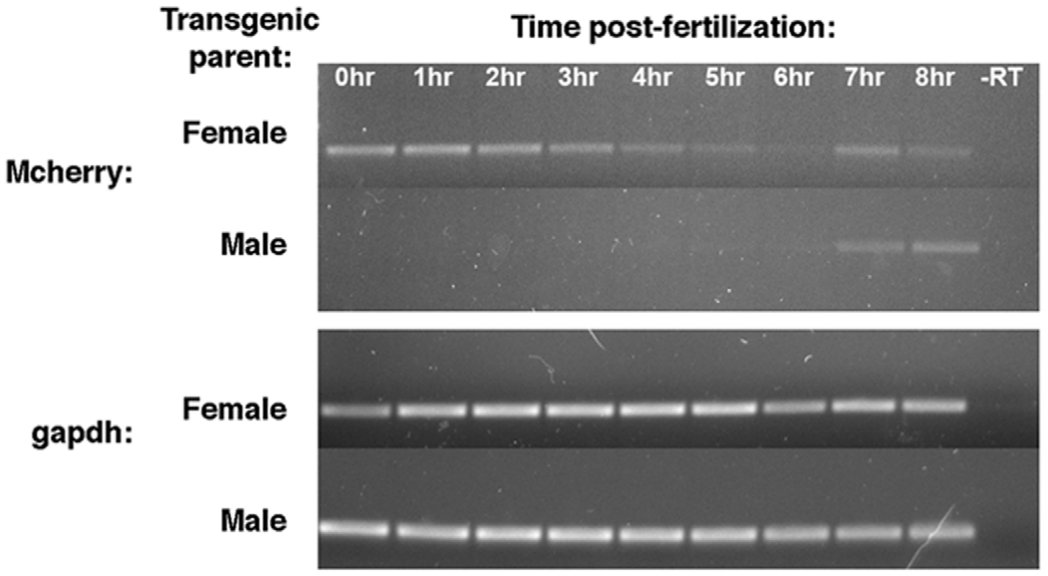
Early expression of the mCherry reporter in transgenic embryos. RT-PCR analysis of *mCherry* from offspring of female or male Tg(Gli-d:mCherry) and wild type adults at stages ranging from 0.25 hpf up to 8 hpf. Expression of *gapdh* served as a cDNA loading control.

To confirm that Tg(Gli-d:mCherry) progeny inherit the transgene, Tg(Gli-d:mCherry) fish were outcrossed with wild type EK or Tu adults. The resulting offspring were probed for *mCherry* RNA expression by RT-PCR. We detected *mCherry* transcripts following fertilization, in the offspring of female Tg(Gli-d:mCherry) that had been mated with male wild type fish (Figure 2). This expression declined slightly around 5 hpf, before increasing again at 7 hpf. On the other hand, *mCherry* transcripts did not appear until 7 hpf in the offspring of male Tg(Gli-d:mCherry) and female wild type matings (Figure 2). This indicated that the mCherry reporter was maternally deposited by Tg(Gli-d:mCherry) females and that zygotic transcription of the transgene commenced by approximately 7 hpf. When these samples were probed for *gli1*, it was also found to be present at fertilization and to be maintained, presumably due to maternal contributions and subsequent zygotic expression (data not shown).

**Figure 3:**
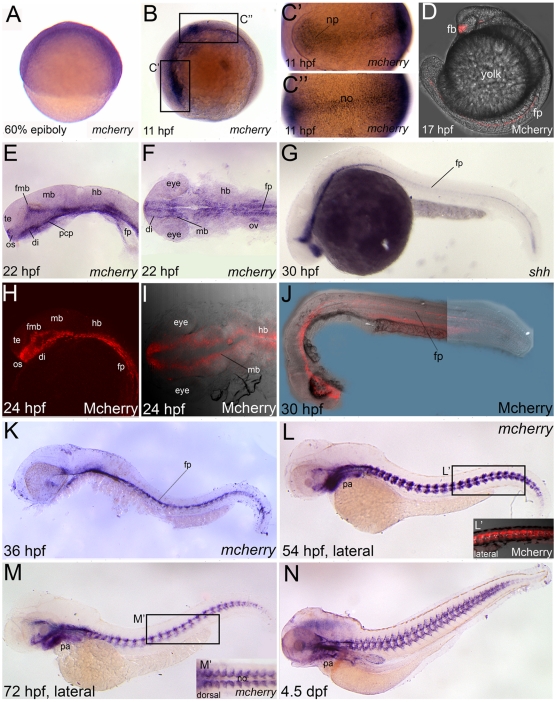
mCherry reporter gene and protein expression reflects known Hh-expressing domains in Tg(Gli-d:mCherry). Lateral views (A,B,D,E,G,H,J,K,L,L′,M,N) or dorsal views (C′,C″,F,I,M′), anterior to the left, of Tg(Gli-d:mCherry) fish labeled with *mCherry* (A–F,K,L,M,N) or *shh* (G) riboprobes or mCherry protein which was visualized by confocal microscopy (H–J,L′). (A) *mCherry* is broadly expressed in blastomeres at 60% epiboly. (B–C″) By 11 hpf, *mCherry* was localized to the neural plate (np) and the midline notochord (no) tissues. (D) At 17 hpf, mCherry protein was apparent in forebrain (fb) and floor plate (fp). (E–G) At 22–24 hpf, *mCherry* gene (E,F) and protein (H–J) are expressed in the fb, midbrain and fp, similar to *shh* gene expression (G) at a comparable stage. (K–N) At later stages from 2 to 4 dpf, *mCherry* is shown in whole-embryo lateral views and detectable in brain, fp, myotomal adaxial cells and PA. (L′) Confocal projections of Mcherry protein corresponding to boxed region in panel L revealed expression in the adaxial cells. (M′) Dorsal view of boxed region in panel M showed *mCherry* expression in adaxial cells and in somitic tissue neighboring the notochord in an orderly, segmental fashion. Abbreviations: di, diencephalon; fb, forebrain; fmb, forebrain-midbrain boundary; fp, floor plate; hb, hindbrain; mb, midbrain; np, neural plate; no, notochord; os, optic stalk; ov, otic vesicle; PA, pharyngeal arches; pcp, prechordal plate; te, telencephalon.

We sought to determine that the Gli-d reporter is expressed in a spatially restricted fashion in Tg(Gli-d:mCherry) fish, reminiscent of the expression pattern for *gli1* and *ptch1*, two genes which are regulated by Gli activity. Spatiotemporal expression of the reporter in Tg(Gli-d:mCherry) was analyzed throughout embryonic and early larval development by both *in situ* hybridization and confocal laser scanning microscopy. *mCherry* gene expression was evident in embryos during epiboly stages, however, at these times the signal was ubiquitously expressed at a low level in most blastomeres (Figure 3A), presumably due to maternal contributions (see above, Figure 2). *mCherry* gene expression first became visible in a spatially restricted fashion within the prospective anterior neural plate and axial mesoderm by the tailbud stage (11 hpf), consistent with the onset of zygote expression a few hours prior to that timepoint (Figure 3B–C″). This expression pattern is consistent with the expression of both *shh* and *gli1* expression patterns [14], [16], [52]. At similar stages, we were unable to detect mCherry proteins by confocal microscopy in Tg(Gli-d:mCherry) embryos (data not shown). Failure to detect a fluorescent signal may be due to low expression levels or decreased protein stability in early-staged embryos. Despite this, by mid-somitogenesis, mCherry fluorescent protein became readily apparent in the embryonic forebrain and within the midline axis (Figure 3D), which also express *shh* and *gli1* at these stages [15], [52]. At 1 dpf (22–30 hpf), lateral and dorsal views revealed mCherry gene and fluorescent protein expression in the forebrain, forebrain/midbrain boundary, floor plate, axial mesoderm tissues and the otic vesicle (Figure 3E,F,H–J), consistent with *shh* (Figure 3G) [15].

Studying *mCherry* expression at later stages, from 36 hpf up to 4.5 days post fertilization (dpf), revealed dynamic expression patterns within the developing brain, pharyngeal arches (PA) and somitic myotome (Figure 3K–N′). With respect to the brain, by 36 hpf *mCherry* expression had became restricted to ventral sites (Figure 3K). Ventral restriction in the central nervous system, after the first day of development, is similar to what has been shown for *gli1* (Figure S2), and for *ptch1* by other groups [15], [53]. Within the developing trunk of 54 hpf embryos, lateral views reveal *mCherry* gene and fluorescent protein expression within the myotome (Figure 3L,L′). By 72 hpf, expression persisted in neural, facial and somitic muscle tissue throughout the larvae (Figure 3M). At the same time, expression in the trunk became more spatially restricted to the population of myotome cells most proximal to the midline notochord, which secretes Hh-ligands at high levels. This reporter expression pattern in the myotome could be best visualized in dorsal views of larval trunks (Figure 3M′). Later, at 4.5 dpf, lateral views of the trunk revealed that *mCherry* extends along the anterior-posterior axis of the developing spinal cord and its expression is spatially restricted within each somite at the prospective sites of intervertebral discs (Figure 3N).

### Changes to the Hh-signaling pathway mediates reporter gene expression in Tg(Gli-d:mcherry) fish

**Figure 4:**
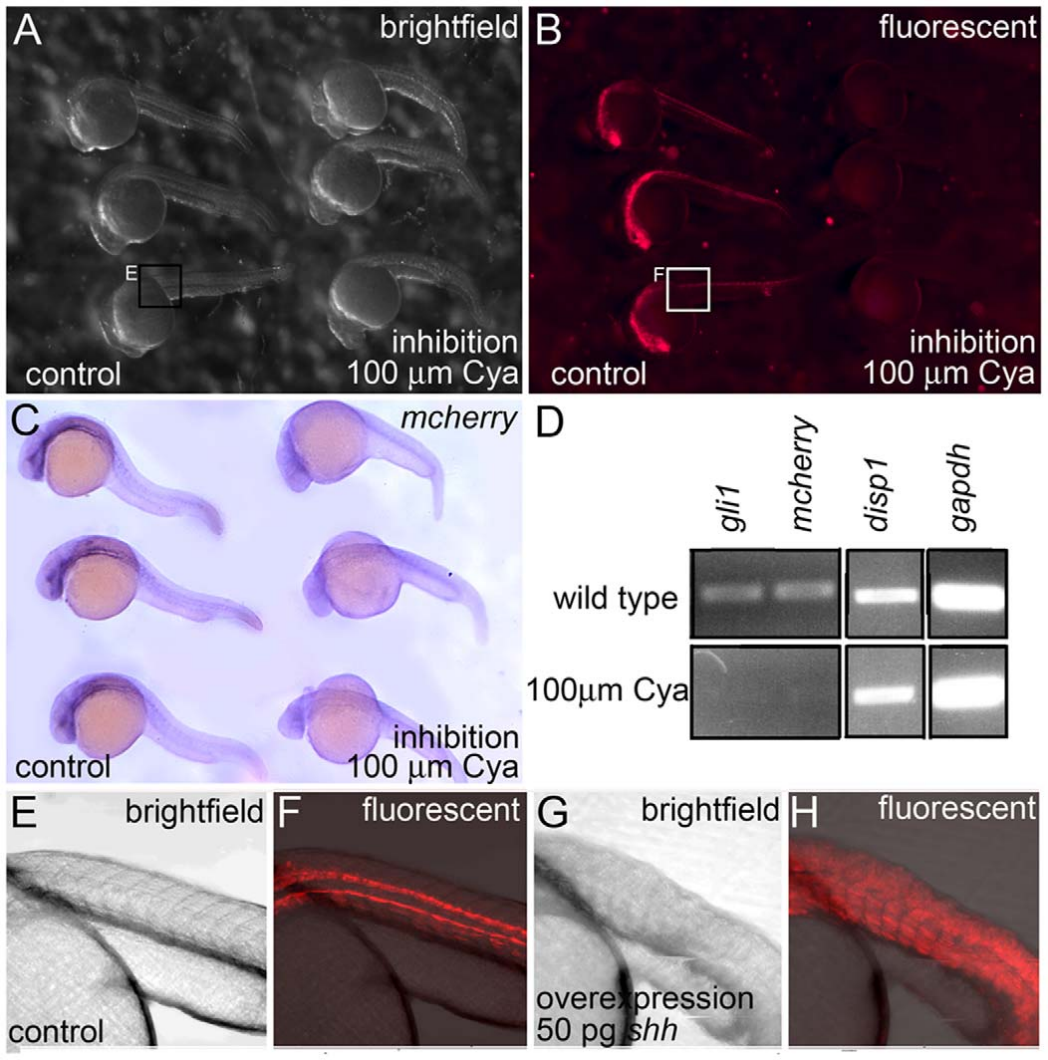
Reporter expression in Tg(Gli-d:mCherry) fish is sensitive to the Hh-signaling pathway. (A–C) Lateral views, anterior to the left, of 30 hpf ethanol vehicle treated controls (left side of image) and Cya treated (right side) Tg(Gli-d:mCherry) siblings. (A,B) Live embryos were visualized using brightfield (A) and fluorescence (B) microscopy. 100 µm Cya treated embryos showed a significant reduction in fluorescent mCherry protein when compared to their ethanol vehicle treated siblings. (C) Fixed embryos, stained with *mCherry* riboprobe revealed that 100 µm Cya treated embryos displayed significantly reduced *mCherry* RNA transcripts compared to their vehicle-treated siblings. (D) RT-PCR showing reduced *gli1*, and *mCherry* expression in 30 hpf embryos treated with 100 µm Cya compared to their nontreated (NT) siblings. Two loading controls for *disp1* and *gapdh* were included. (E–H) Lateral views of 30 hpf noninjected controls (E,F) or Tg(Gli-d:mcherry) siblings injected with 50 pg *shh* at the one cell stage (G,H). Siblings injected with *shh* mRNA showed expanded reporter expression throughout the trunk when compared to noninjected controls.

To confirm the Hh-sensitivity of the stable transgenic fish, we in-crossed Tg(Gli-d:mCherry) fish and then incubated the offspring with 100 µM Cya from dome stage to 30 hpf. Following Cya treatments, or similar treatments with a vehicle control (ethanol), we screened half of the embryos live to visualize mCherry protein fluorescence, while the remaining embryos were fixed and screened for *mCherry* RNA expression by *in situ* hybridization. Among vehicle-treated transgenic embryos, fluorescence was apparent in the forebrain and trunk within the majority of live anesthetized embryos (32/42 embryos displayed fluorescence) (Figure 4A,B). In contrast, fluorescence was never detected in trangenic siblings treated with Cya (0/20 embryos displayed fluorescence) (Figure 4A,B). Additionally, *mCherry* RNA transcripts were suppressed in a similar fashion to fluorescent mCherry protein in Cya treated embryos (0/20 embryos displayed fluorescence), while vehicle treated control siblings displayed normal gene expression patterns (26/38 embryos displayed fluorescence) (Figure 4C). Transgene gene expression levels were further analyzed by RT-PCR. Using the same conditions as described above, RNA was extracted from whole embryos and Hh-sensitive (*gli1*) and insensitive (*disp1*, *gapdh*) genes were assessed along with *mCherry*. We found that *mCherry* expression was as sensitive to Hh-signaling inhibition as *gli1* (Figure 4D).

We next sought to determine if Tg(Gli-d:mCherry) fish could respond to cellular increases in Hh-signaling. Ectopic Hh-signaling is sufficient to activate *Gli1* expression in mouse, chick, frog and zebrafish [7], [15], [33]–[36]. To increase the embryonic levels of Shh protein in transgenic embryos, *in vitro* transcribed *shh* mRNA was injected into one cell stage Tg(Gli-d:mCherry) fish. At 30 hpf, *shh* injections consistently resulted in an expansion of mCherry fluorescent protein expression, beyond the normally restricted expression patterns within the head (data not shown) and trunk regions that we have reported for Tg(Gli-d:mCherry) (Figure 4 E–H). In full, these results indicate that the Tg(Gli-d:mCherry) fish lines generated in this study express the mCherry reporter under the control of the Hh-signaling pathway.

### Expression of the Gli-dependent reporter in craniofacial region of transgenic fish

The zebrafish craniofacial skeleton is comprised mostly from CNC cells. Multipotent CNC cells originate in the dorsal midbrain and hindbrain, from where they delaminate and subsequently migrate ventrally into the zebrafish head. Once they have migrated into the jaw and PA, they intricately associate with facial epithelium (both ectodermal and endodermal) which provide structural support and also secrete molecules that drive differentiation of CNC into cartilage [54], [55]. Our previous studies, as well as work from others, revealed that Hh-ligands are expressed in a dynamic fashion during craniofacial development, in the brain, facial ectoderm and facial endoderm [41], [42], [47]. During the late pharyngula stage (32–48 hpf), Hh-ligands are secreted from at least one of these tissues and are required to induce the differentiation of postmigratory CNC cells in the posterior arches (PA 3–7) to chondrocytes [42]. Despite this knowledge, we currently have a limited understanding of which cells in the head are responding to the available Hh-signals during PA chondrogenesis. The Tg(Gli-d:mCherry) represents a useful tool for determining the spatiotemporal pattern of Gli activity within specific cellular domains of the craniofacial region during skeletal development.

As we began our studies, we speculated that the CNC mesenchyme represented the likeliest targets of Hh-signals during the late pharyngula stage. To test this hypothesis, Gli-dependent reporter activity was carefully examined in the craniofacial tissues of Tg(Gli-d:mCherry) fish, beginning with the late pharyngula stage. Concurrently, we examined the expression of each *gli* gene (*gli1*, *gli2a*, *gli2b*, *gli3*) within craniofacial tissues, whose collective expression patterns represent both potential activator and repressor activity on Hh-target genes. For the majority of our analysis of Gli-dependent reporter expression in craniofacial tissues, we assessed mRNA expression which likely reflects changes to the Hh-pathway more sensitively than protein as regulation of the pathway occurs at the level of transcription. Moreover, it was important to use fixed tissue in these studies as clear views of the pharyngeal region required dissection of yolk cells, and in some cases, removal of the eye.

**Figure 5:**
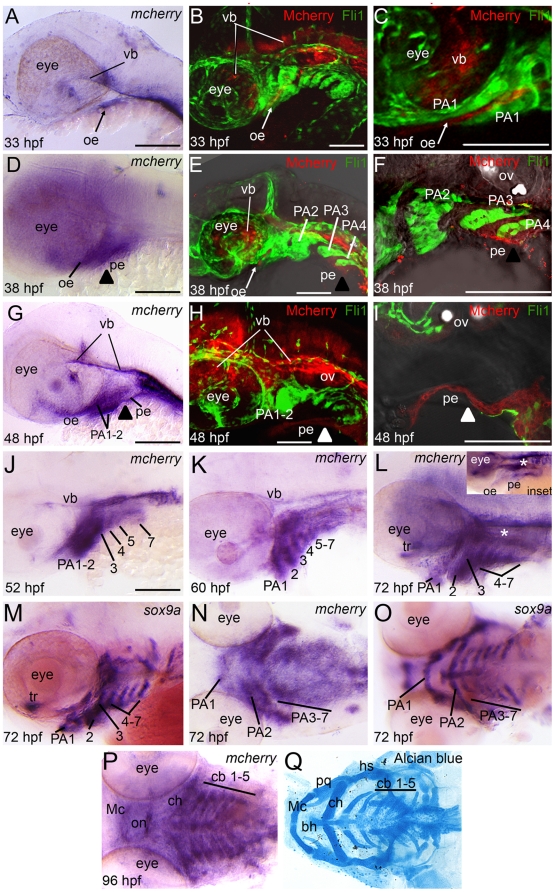
Reporter expression in Tg(Gli-d:mCherry) craniofacial region during embryogenesis and larval stages. Lateral (A–M) or ventral (N–Q) views, anterior to the left, of 33 hpf (A–C), 38 hpf (D–F), 48 hpf (G–I), 52 hpf (J), 60 hpf (K), 72 hpf (L–O) or 4.5 dpf (P,Q) transgenic fish stained with *mCherry* riboprobe (A,D,G,J,K,L,N,P) or *sox9a* riboprobe (M,O) for gene expression or Alcian blue (Q) to visualize cartilage elements. Confocal stack projections of Tg(Gli-d:mCherry) and Tg(Fli1:gfp) double transgenic fish (B,C,E,F,H,I). In these fish, reporter (red) and neural crest cells (green) can be visualized simultanenously. Of note, in (H–I) the reporter-positive pe tissue separated slightly from PA tissue during mounting of embryo. In (I) green-positive neural crest cells are not in the focal plane, despite residing just dorsal to mCherry expressing pe cells. (A–C) At 33 hpf, transgene is expressed in ventral brain and oral ectoderm. (D–F) At 38 hpf, expression persists in brain and ectoderm and expands to pharyngeal endoderm. (G–I) At 48 hpf, expression persists in brain and facial epithelium and expands to mesenchyme in anterior arches. (J–K) From 52–60 hpf, transgene expression expands to mesenchyme in all arches. (L–O) At 72 hpf, transgene expression in PA closely matches the mesenchymal neural crest marker *sox9a*. (P,Q) At 4.5 dpf, transgene expression persists in each arch and the pattern matches cartilage elements. Abbreviations: bh, basihyal; cb, ceratobranchial; ch, ceratohyal; hs, hyosymplectic; Mc, Meckel's cartilage; oe, oral ectoderm; on, optic nerve; PA, pharyngeal arch; pe, pharyngeal endoderm; pq, palatoquadrate; tr, trabeculae; vb, ventral brain.

Expression of *shh*, the major Hh-ligand, in craniofacial tissues during development is primarily restricted to the ventral brain from late somitogenesis up to 33 hpf at which point a new expression domain becomes apparent in the facial ectoderm and endoderm [42], [47]. Similar to *shh* expression, *mCherry* and *gli* expression in the Tg(Gli-d:mCherry) fish was mainly localized to the brain prior to 33 hpf (see Figure 3E–G, Figure S2). However, at 33 hpf a new *mCherry* expression domain became apparent in the facial tissue (Figure 5A). To better determine the identity of the *mCherry* expressing cells in the face, Tg(Gli-d:mCherry) fish were outcrossed to the Fli1-GFP transgenic zebrafish line which express GFP in postmigratory CNC cells [56]. Transgenic embryos from this mating allowed us to simultaneously visualize Hh-responding domains (red, mCherry) and head CNC (green, GFP). These embryos revealed that Gli activity in the facial tissue at 33–34 hpf is spatially restricted to the oral ectoderm (oe), based on its intricate association with first arch neural crest mesenchyme (Figure 5B,C, arrow). During this time, reporter expression was also localized to the ventral brain (vb) (Figure 5A–C, asterisks denote ventral brain). Similar to *mCherry* reporter expression at 33 hpf, *gli1* and *gli2a* was also expanded from neural tissue alone to facial tissues (Figure S2). Later, at 38 hpf, the reporter gene and protein remained strongly expressed in the oe, despite being expressed at reduced levels in the brain (Figure 5D–F). Interestingly, at 38 hpf a new expression pattern emerged in the pharyngeal endoderm (pe) tissue (Figure 5D, arrowhead). Closer examination with confocal microscopy on double transgenics clearly showed reporter expression in the pharyngeal endoderm surrounding the CNC mesenchyme (Figure 5E,F). The reporter protein was localized primarily to endoderm within the third through fifth arches in the transgenic fish shown (Figure 5F). Next, at 48 hpf, the reporter gene remained expressed in the brain, facial ectoderm and endoderm (Figure 5G), similar to the expression pattern for *shh* at this stage [42], and also closely resembling the expression pattern for *gli1* (Figure S2). Consistent with reporter gene expression, mCherry protein was clearly present in the facial endoderm and facial ectoderm at these stages (Figure 5H,I, Figure S3). At this stage, we also noticed expanded gene expression in the zebrafish jaw, potentially corresponding to CNC mesenchyme in the anterior-most PA (Figure 5G). Similarly, *gli* transcripts, especially *gli2s* (*a* and *b*) but also *gli3*, became expanded in the face at 48 hpf (Figure S2), however these genes were more broadly expressed in the jaw and other facial tissues than the reporter. Due to the balance of activator and repressor forms of gli proteins, it is likely that the expanded expression of multiple *glis* at this stage refines the Hh-signal, as has been shown in other developmental contexts [13], [15], [18], [19], [25], [57]. In a similar vein, at this timepoint and all earlier stages, we never detected Gli-dependent reporter expression in the CNC mesenchyme within the posterior arches, strongly suggesting that the CNC mesenchyme in these arches are not actively receiving or responding to Gli activation during these stages of development, despite our previous findings that CNC mesenchyme fails to differentiate if the pathway is attenuated during this time (specifically during 32–48 hpf). Rather, expression of the Gli-dependent reporter at these developmental stages closely resembles known Hh-ligand expressing domains in the brain and facial tissues that neighbor the postmigratory CNC mesenchyme in the posterior arches.

While reporter expression was not detectable in posterior arch CNC mesencyme during the first two days of development, by 52–60 hpf *mCherry* RNA transcripts became visible in the CNC mesenchymal condensations within each PA (Figure 5J,K). By 72 hpf, *mCherry* was strongly expressed in nearly all ventral facial cartilage precursor cells as visualized in lateral (Figure 5L) or ventral views (Figure 5N). Consistent with reporter expression being in the CNC mesenchyme at this stage, the expression pattern of *mcherry* was nearly identical to the chondrogenic marker *sox9a*, a CNC marker that controls chondrification (Figure 5M,O; [58]). In addition to its expression in the CNC cells, *mCherry* remained detectable in the brain and pharyngeal endoderm at 72 hpf (Figure 5L, inset shows image with endoderm in focal plane [CNC cells out of focus]), similar to *shh* expression at this stage [42,42]. Expression of *gli* genes at concurrent stages of development provided further support for Hh-signaling targeting the CNC mesenchyme at later stages, as expression of *gli1* and *gli2a* remained highly expressed in the facial tissues after the second day of development and both genes appeared strongest in the mesenchyme at 72 hpf (Figure S4). Finally, at later stages (4.5 dpf), *mCherry* continued to be expressed in CNC cells after their differentiation into cartilage (Figure 5P), at which point the ventral pharyngeal cartilages were completely formed (Figure 5Q).

In summary, mCherry reporter expression was dynamic in craniofacial tissues during embryonic and early larval development, first detectable in the ventral brain from 1 dpf where it remained detectable up to 4.5 dpf. Reporter expression was also detected in the facial ectoderm and endoderm from 33–38 hpf where it remained visible up to 4.5 dpf. Finally, we saw reporter expression in the CNC cell jaw precursors, from 48 hpf up to 4.5 dpf. Unlike jaw cartilage precursors, CNC that will become the cartilage elements in the posterior arches express the reporter beginning around 52–60 hpf and continue to express it up to 4.5 dpf.

### Hh-signaling controls PA chondrogenesis by its influence on the brain or facial epithelium

Our expression analysis on Tg(Gli-d:mCherry) fish showed that posterior-arch residing CNC are not directly targeted by Hh-signaling during the late pharyngula stage (32–48 hpf); however, Hh-signaling is highly active in other craniofacial tissues during this stage, as transgene expression was visible in neural tissues and facial epithelium. Since reporter expression did become apparent in CNC mesenchyme shortly after the late pharyngula stage, we wondered if blocking Hh-signaling during the late pharyngula stage may either reduce the availability of Hh-ligands from surrounding neural or facial tissues to the postmigratory CNC mesenchyme at later stages, and/or make the CNC mesenchyme less capable of responding to Hh-signals after the late pharyngula stage.

**Figure 6:**
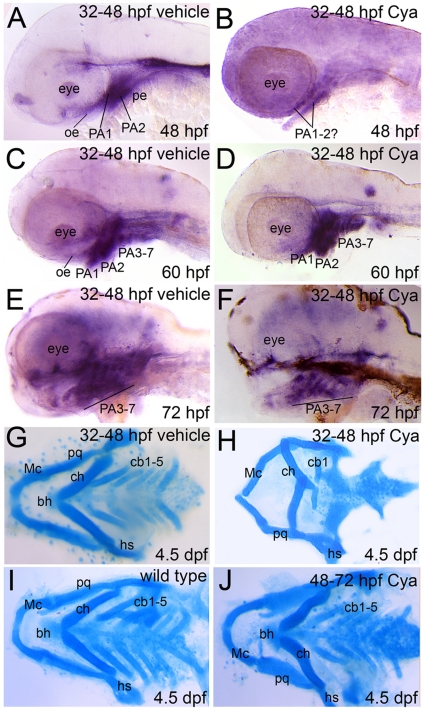
Reduced Gli activity in craniofacial tissues following late pharyngula stage Hh-pathway inhibition. Lateral (A–F) or dorsal (G–J) views, anterior to the left, of Tg(Gli-d:mCherry) fish treated with vehicle (A,C,E,G), Cya (B,D,F,H,J) or not treated (I) and stained with *mCherry* riboprobe (A–F) or Alcian blue solution (G–J). (A,B) Reporter gene expression was significantly reduced in the brain and facial epithelium at 48 hpf following late pharyngula stage Cya treatment. A slight *mCherry* signal was detected in craniofacial tissue that likely corresponds to PA1-2 mesenchyme at 48 hpf following Cya treatment. (C–F) Reporter gene expression was fully recovered in Cya treated embryos by 60–72 hpf and is expressed in oe, pe, brain and PA mesenchyme. (M–O) Alcian blue staining to reveal cartilage deficits in Gli reporter transgenic fish following Cya treatments at 32–48 hpf (H) or 48–72 hpf (J). These deficits are consistent with our previously published findings and indicate that Cya reliably inhibited cb chondrofication upon Hh-inhibition during the late pharyngula stage (H), but did not negatively impact cartilage development when administered at later stages (J).

To inhibit Hh-signaling during the late pharyngula stage, embryos were treated with Cya from 32 hpf until 48 hpf, at which time Hh-signaling was restored by washing away the Cya solution and allowing development to proceed normally. This Cya treatment protocol was chosen as it was during this developmental stage, using the same treatment protocol, that we previously identified Hh-signaling as being critical for chondrogenesis in posterior arches [42]. At 48 hpf, sixteen hours after Hh-inhibition began, Tg(Gli-d:mCherry) embryos continued to display strong transgene expression within tissues corresponding to the anterior arches, suggesting that this Cya treatment protocol did not abolish the *gli*-dependent response in these cells (Figure 6A,B). Reporter expression in these tissues is not likely due to persisting mRNA transcripts, produced at stages prior to inhibition, since we have found that both *mcherry* reporter transcripts and *gli1* transcripts begin to decline at four hours of starting a Cya inhibition treatment (100 µm) and are completely reduced by six hours, as assessed by real time PCR (data not shown). Despite some reporter expression perduring in the anterior arches, transgene expression in the brain, oe, pe (Figure 6B) and trunk (data not shown) was reduced by inhibition. This indicated that late pharyngula stage inhibition reduced nearly all of the Hh-response in the brain and facial epithelium which surround cartilage precursors during this developmental stage. By 60 hpf, transgene expression had completely recovered in all tissues that normally respond to Hh-signaling at this stage. Reporter expression could be detected in neural, facial epithelium and CNC mesenchymal condensations in each arch (Figure 6C,D). Similarly, transgene expression persisted in CNC mesenchyme at 72 hpf (Figure 6E,F) and up to 4.5 dpf (data not shown), despite the fact that CNC cells in the posterior arches failed to chondrify in these larvae (Figure 6G,H). This data clearly shows that late pharyngula stage inhibition does not negatively impact the ability of CNC mesenchyme to receive Hh-signals at later stages, despite their reduced ability to differentiate properly. We have shown previously using similar timed inhibition studies that inhibiting Hh-signaling any time after the late pharyngula stage does not impact cartilage development in the PA [42]. These findings collectively suggest that direct Hh-signaling, mediated by Gli, is not required by CNC to differentiate into cartilage.

In full, this data supports a conclusion that Hh-signaling during the late pharyngula stage must be received by neural or facial tissues. Reception of the signal in these cells, which directly neighbor the cartilage precursors of the ventral head skeleton, may lead to the downstream production and secretion of an unknown, secondary signal required by the cartilage precursors to maintain expression of chondrogenic factors. More work will be required to determine the targets of Hh-signaling in the neural and facial tissues that may represent potential secondary signals in PA chondrogenesis.

### Gli activity in craniofacial tissues in the absence of Hh-signaling

**Figure 7:**
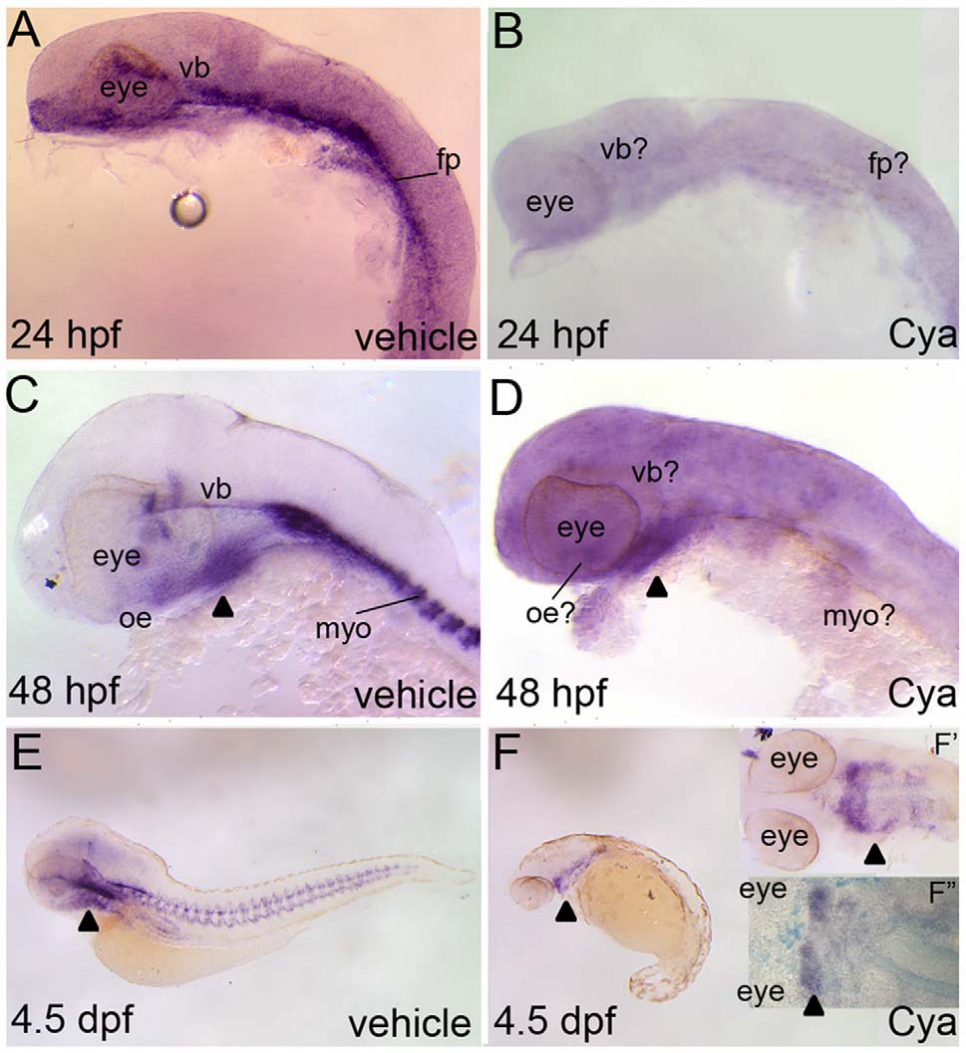
Reporter expression in Tg(Gli-d:mCherry) fish following severe reductions to Hh-signaling. Lateral (A–F) or ventral (F′,F″) views, anterior to the right, of Tg(Gli-d:mCherry) fish treated with vehicle (A,C,E) or Cya (B,D,F,F′,F″) and stained with *mCherry* riboprobe (A–F′) or dually stained with *mcherry* and Alcian blue (F″). Vehicle or Cya treatments began at dome stage and commenced just prior to sacrificing the embryos or larvae for staining analysis. (A,B) At 24 hpf, Cya treatments suppressed all head and trunk reporter expression in transgenic embryos. (C,D) At 48 hpf, Cya treatments reduced all reporter expression in transgenic fish, with the exception of a small expressing domain in the ventral head (arrowhead). (E,F) Reporter expression was virtually absent in Cya treated 4.5 dpf transgenic larvae, with the exception of the same *mCherry* expressing domain in the ventral head that was seen in 48 hpf aged embryos. (F′, F″) Closer examination of the ventral head suggested that the *mCherry* expressing cells are within second arch mesenchyme and that no cartilage forms in this arch or any other PA in Cya treated larvae. Abbreviations: fp, floor plate; myo, myotome; vb, ventral brain.

One surprising outcome of our reporter expression analysis in Tg(Gli-d:mCherry) fish was the finding that a population of cells in the anterior arches of 2 day old embryos continued to express the Gli reporter transgene despite abrogating the Hh-pathway with Cya in these embryos (see Figure 6B). While Hh-signaling has been shown to be necessary for normal transcription of *gli* genes in zebrafish and mouse [59], it has been previously reported that very weak expression of *gli1* remains detectable in the absence of Hh-signaling in the zebrafish [15], at least up to 20 hpf. Since the reporter in Tg(Gli-d:mCherry) reveals the cellular sites of Gli activity, not Gli transcription outputs, we wanted to identify any peristent Gli activity in Hh-compromised fish. To achieve this, Tg(Gli-d:mCherry) fish were treated with Cya throughout development, from dome stage (4 hpf) up to the timepoint at which the fish were fixed and reporter expression was assessed at 24 hpf, 48 hpf, and 96 hpf. As shown previously in this report (Figure 4), treating Tg(Gli-d:mCherry) embryos with Cya during the first day of development completely abrogated all reporter expression in the embryo when examined at 24-28 hpf. Closer examination of head and Tg(Gli-d:mcherry) brain tissues following Cya inhibition confirms the complete reduction of Gli activity in treated embryos (Figure 7A,B). Contrary to this, when reporter expression was analyzed in Cya treated Tg(Gli-d:mCherry) that were 48 hpf (Figure 7C,D) or older (Figure 7E,F), a group of reporter expressing cells were clearly evident in the anterior arches. The persistent *mCherry* expression domain in the lower jaw was quite noteworthy, as the Cya treatment sufficiently eliminated reporter expression throughout all other areas of the larvae (Figure 7F). Moreover, larvae treated from 4–96 hpf displayed phenotypes characteristic of severe Hh-signaling inhibition, including U-shaped somites, a ventrally curled tail, heart edema and mild-to severe cyclopia. A closer examination of these larvae indicated that the remaining *mCherry* expression domain was likely localized specifically to the second PA mesenchyme, although the transcripts were expressed in a highly diffuse fashion, at a low level, in more posterior areas of the pharyngeal region (Figure 7F′). Staining these larvae with alcian blue revealed that no cartilages formed upon complete Hh-inhibition with Cya (data not shown), similar to previous reports on *smu/smo* genetic mutants [39]. To be sure that Cya inhibition in these larvae was not completely eliminating both head cartilages and *mcherry* expressing cells in the head, we dually stained some Cya treated larvae for both *mcherry* expression and Alcian blue expression. In these fish, we consistently noticed *mcherry* expression, even in the absence of any cartilages (Figure 7F″).

Collectively this data suggests that a small population of cells in the ventral zebrafish head retain Gli activity in the absence of Hh-signaling. While the Gli-d reporter appears to be expressed in second arch mesenchyme, any potential Gli activity in these cells does not impact craniofacial cartilage development, consistent with our findings herein that CNC progenitors do not directly require Gli activity to differentiate to cartilage cells. While we cannot rule out the possibility that their exists low levels of reporter expression in Tg(Gli-d:mCherry) that is independent of Gli activity, our findings here are consistent with those of Karlstrom et al., 2003 where it was shown that some *gli1* transcripts become made in zebrafish embryos where the Hh-pathway has been fully compromised [15]. This may be unique to the fish, as Gli1 expression is completely absent in *Smo* mutant mice [59]. Moreover, reports on *gli1* acting independently of the Hh-signaling pathway (i.e. in a non-canonical fashion) are few in vertebrates [60] and are most often associated with tumor environments.

## Discussion

The purpose of the experiments in this report was to create a transgenic zebrafish line that would allow for *in vivo* visualization of cells that are responding to Hh-signaling using Gli-activity as a read-out for the pathway. mCherry reporter expression in Tg(Gli-d:mCherry) embryos was spatially restricted adjacent to *shh*-expressing domains, most notably the ventral central nervous system, trunk myotome and craniofacial tissues. Moreover, the reporter was expressed in identical or neighboring tissues that expressed *gli* genes in the craniofacial tissues, further arguing that it is responsive to Gli activity and likely reflects a balance of dual Gli promoter and repressor activity. The reporter protein could be visualized in live or fixed tissue by fluorescent microscopy without the use of antibody amplification, allowing for rapid signal detection. Being able to detect fluorescence changes in live embryos, as a result to changes to the Hh-pathway, will be beneficial in assessing in real-time the ability of a specific compound or gene product, applied topically by water-delivery or by microinjection, to alter the pathway. Furthermore, by generating an antisense RNA riboprobe for the ORF of *mCherry*, the reporter RNA transcripts can also be detected in a highly sensitive fashion by *in situ* hybridization. Detection of mRNA reporter transcripts in fixed tissues will allow researchers to directly visualize Hh-response in conjunction with other cell markers, as assayed by *in situ* hybridization or immunohistochemistry, as well as markers for cell proliferation or cell death.

Using Hh-signaling genetic mutants as well as treatments involving a pharmacological inhibitor for Hh-signaling, we could determine that the reporter element was sensitive to Hh-signaling inhibition *in vivo*, when stably expressed in Tg(Gli-d:mCherry) or when the reporter DNA plasmid was transiently expressed in wild type embryos by microinjection at the one cell stage. Further, the reporter element was also responsive to ectopic expression Hh signaling, as expression of Shh DNA led to expansion of mCherry expression beyond the spatially restricted domains. While reporter expression in Tg(Gli-d:mCherry) resembles known *gli1* expressing domains, we can not rule out the possibility that some Hh-responding domains are not represented by mCherry expression in the transgenic line. Certain cell population may display restricted transgene expression due to local chromatin effects or other epigenetic factors. It will require further examination of future reporter-expressing, transgenic lines to rule out this possibility.

Insight about the nature of Hh-signaling can be gained through analyzing the Tg(Gli-d:mCherry) under normal developmental conditions, or when the Hh-signaling pathway is experimentally modulated. In experiments reported in this study, we were able to detect changes to the Hh-signaling upon treating embryos with the pharmacological Hh-inhibitor Cya. Reductions to the Hh-signaling pathway on treated embryos could be easily detected when the reporter DNA construct was transiently expressed by microinjection into wild type embryos or stably expressed in transgenic embryos. Concurrent with our study, Yang et al., recently introduced a similar Gli-dependent transgenic line that contains a GFP reporter which is strongly expressed in the developing somites but is inhibited when the fish are ectopically treated with water soluble Hh inhibitors [61]. Our findings in this study support the use of these transgenic lines as a tool to rapidly screen chemical compounds. Additionally, we have shown here that the Tg(Gli-d:mCherry) fish represent an important line that can be utilized to report on Hh-signaling during disease and development.

The Tg(Gli-d:mCherry) zebrafish should allow Hh-signaling to be studied in a variety of embryonic or adult tissues. We designed the fish in an effort to more precisely detect Hh-responding cells in the craniofacial region during embryonic and larval development. We have previously shown that Hh-signaling controls ventral arch cartilage development by impacting CNCC patterning and differentiation [42]. It is well known that Hh-ligands are expressed in a dynamic fashion in the craniofacial tissues during development; primarily within the ventral brain up to 33 hpf, wherein *shh* expression domains expand to facial ectoderm and endoderm. Missing from this analysis was which cell populations in the brain and face are receiving the Hh-signal at synonymous timepoints.

In this report, we have characterized the reporter expression in the craniofacial tissues of the Tg(Gli-d:mCherry) line throughout development to provide a detailed analysis of Hh-responding domains in these regions. To summarize the data, we found that Hh-signals are received by neural tissues, predominantly in the ventral brain, from somitogenesis stages up to 4.5 dpf. Facial ectodermal cells began expressing the reporter at 33 hpf, while reporter expression in the pharyngeal endoderm began around 36–38 hpf. At these stages, CNCC have completed their migration from the neural tube into the arches and signals from the facial epithelium are likely required to by the neural crest mesenchyme to become properly patterned. Hh-signals received by the facial epithelium cannot originate in the CNCC, as Hh-ligands are not expressed in these cells at this time. The likeliest sources of the Hh-signals to the facial epithelium are the ventral brain, or facial epithelial cells signaling in an autocrine fashion. Reporter expression could be detected in the ventral brain, facial ectoderm and endoderm up to 72 hpf.

With respect to reporter expression in the neural crest mesenchyme, we found that the first population of cells within the PA to express the reporter was post-migratory mesenchymal condensations within the lower jaw at 48 hpf. Shortly thereafter at 52 hpf, nearly all neural crest mesenchymal condensations within the PA expressed the transgene. Reporter expression could be visualized in PA mesenchymal condensations at timepoints beyond their chondrification to cartilage cells. The potential sources of the Hh-signals to the CNCC may be ventral brain or facial epithelium and more experimentation will be required to delineate the origin of the signal.

Reporter expression in the neural and facial tissues at each developmental stage examined was further supported by expression analysis of all identified zebrafish *gli* genes. Collectively, these four genes represent protein products that may activate or repress Hh-target genes. Since our reporter is most likely reflects the combination of positive and negative Gli activity functions, it was not surprising to find that its expression closely resembled the pattern for each *gli* gene. Overall, reporter expression could be characterized as less broad and more refined to specific tissue groups (i.e. the pharyngeal endoderm or neural crest mesenchyme) than the collective expression of *gli* genes. The finding that the reporter was expressed in a fine-tuned pattern at each stage of craniofacial development is in line with what one may have predicted for the readout of such a critical signaling pathway.

Upon initiation of this study, we hypothesized that Hh signals were directly required by cartilage precursors in the PA to undergo chondrogenesis into the ceratobranchial cartilage elements of the ventral zebrafish skeleton. This hypothesis was driven primarily by earlier evidence that mesenchymal condensations in the posterior arches fail to maintain expression of pro-chondrogenic transcription factors *sox9a* and *dlx2a* in *con/disp* mutants. Further, we had also shown that the requirement for Hh-signaling to maintain normal expression levels in the neural crest mesenchyme was during the late pharyngula stage (32–48 hpf), at which time neighboring neural and facial epitheilial cells robustly express *shh* [42]. However, we have determined that mesenchymal condensations in the posterior arches were not positive for the Gli reporter transgene until approximately 52–60 hpf. Thus, we conclude that Hh-signaling may act by a paracrine mechanism; inducing a mediating molecule to be expressed by the brain or pharyngeal endoderm which would then be received by the CNCC residing in the posterior arches. This potential mediator signal would be sensitive to modulations in Hh-signaling and its action would promote the expression of pro-chondrogenic factors in neural crest residing in the posterior arches.

If posterior-arch residing neural crest cells do not directly require Hh-signaling to undergo chondrogenesis, why then do they robustly express the Gli-dependent reporter from the second day of development and at timepoints up to 4.5 dpf? One potential explanation is that Hh-signaling controls further aspects of facial development, such as the maturation and morphogenesis of chondrocytes and perichondrium undergoing endochondral ossification [62]–[65]. Hh-signaling controls chondrocyte and perichondrium ossification primarily through the expression of *Indian hedgehog* (*Ihh*) genes, which become expressed in maturing chondrocytes and provide signaling cues to both chondrocytes and the surrounding perichondral cells to influence the timing of osteoblast differentiation [65], [66]. In zebrafish, *ihh* genes become expressed in the ceratohyals by 5 dpf [67], before becoming more widely expressed in chondrocytes of the developing pharyngeal skeleton by 6 dpf [68]. Like *shh* ligands, *ihh* genes exert their activity through *gli* genes, which are not surprisingly also expressed in chondrocytes and perichondrium at these timepoints [67]. Expression of these Hh genes is functionally relevant, as it was recently shown that increasing or depleting Hh-signaling in fish negatively influences osteoblast and osteoclast development [69]. In this study, Gli activity was mostly examined prior to *ihh* gene expression in the head cartilages and when *shh* genes are believed to be the primary Hh-ligands being expressed. It is likely, however, that later reporter expression in Tg(Gli-d:mcherry) fish that are 4 days or older will allow for the visualization of Gli activity in maturing chondrocytes and perichondrium due to Ihh expression which will enhance our understanding of Hh-signaling in bone development. Additionally, reporter expression in the facial epithelium and CNC mesenchyme after timepoints required for chondrogenesis may be reflecting a requirement for Hh-signaling in other aspects of facial development, such as the development of cranial muscles. In support of this, we have found that cranial muscle development is perturbed in Hh-pathway genetic mutants (Schwend and Ahlgren, unpublished). This data has been difficult to obtain, as genetic disruptions to the Hh-pathway are often embryonic lethal and death occurs prior to developmental stages where muscles are forming (beyond 5 dpf). However, by utilizing genetic mutants where the Hh-signaling pathway is abrogated to a lesser extent (such as the *con/disp1* mutant) or late-stage Cya inhibition, at timepoints after most vital organs have developed, it is possible to keep larvae alive long enough that facial development may be assessed. Currently, we are in the process of studying the role of Hh-signaling in the development of these cranial tissues. Certainly, the use of the Tg(Gli-d:mCherry) line will be highly fruitful to these continued studies.

One surprising result from our characterization of Tg(Gli-d:mCherry) line was that a domain of *mCherry* expressing cells in the second arch mesenchyme maintained expression despite full pathway inhibition with Cya. Gli transcription factors normally become activated in a canonical fashion, dependent on Hh-ligand presentation and Smo activation. Cya inhibits Hh-signaling at the level of the Smo receptor, and thus *gli1* transcription should not become activated by the Hh-pathway in the presence of Cya. Thus, second PA domain may represent a group of cells in the craniofacial region that may activate *gli1* transcription in the absence of Hh-signaling fashion. It had been previously shown that a very low level of *gli1* transcription does occur in *smu/smo* mutant zebrafish, primarily in anterior tissues, however it had not been known if these sources of Gli could activate downstream targets [15]. Our data suggests that a small amount of Gli activity remains in the absence of Hh-signaling, localized primarily to the jaw after the second day of development. We cannot rule out the possibility that low levels of Hh-signaling, above the threshold for *gli* activation in these cells, exists in the presence of Cya. Furthermore, it remains a possibility that epigenetic factors in this cellular domain of our transgenic fish perturb the ability of the Cya to fully inhibit cells in this region, thus resulting in nonspecific expression of the transgene.

In summary, we have reported on the generation of a transgenic zebrafish line that allows for *in vivo* detection of cells responding to Hh-signaling. This transgenic line can be used to better understand the spatiotemporal pattern of Gli activation in a variety of organ systems in the zebrafish. Furthermore, because the fish line is sensitive to Hh-signaling, the fish can be utilized in research studies designed to test potential therapeutic agents hoping to be used as Hh-signaling agonists or antagonists. Our understanding of the Hh-signaling mechanism in development and disease will ultimately be better understood due to the generation of these transgenic lines.

## Materials and Methods

### Animals

Zebrafish (*Danio rerio*) embryos were obtained from natural crosses and staged as previously described [70]. All experimental protocols involving live animals were approved by the Animal Care and Use Committee of Children's Memorial Research Center in Chicago, Illinois. The Tuebingen (Tu) strain of fish was originally obtained from the Zebrafish International Resource Center (ZIRC) in Eugene, Oregon, and the Ekkwill strain of fish were obtained from Ekkwill Waterlife Resources (Gibsonton, Florida). *detour/gli1* (*dtr* ^ts69^), *you-too/gli2* (*yot^ty119^*), and *slow-muscle-omitted/smu* (*smu* ^b641^) were previously identified in phenotypic screens for mutant phenotypes and their genetic mutations in the Hh-signaling pathway have been subsequently described [14], [15], [38], [39], [71]. Homozygous genetic mutants were identified by somite morphology (U-shaped as opposed to chevron shaped) at 30 hpf as previously described [71]. Tg(Fli*:*gfp) transgenic zebrafish expressing the gfp-transgene under the control of the Fli1 promoter was previously characterized [56].

### Generation of Tg(Gli-d:mCherry) zebrafish

A 765 basepair fragment containing eight repeating Gli-CBS from the mouse FoxA2 floor plate enhancer and a minimal lens crystallin promoter [40] were flanked by Invitrogen Multisite Gateway compatible *att* sites by PCR amplification using a forward PCR primer containing an *attB4* site and template specific sequence and a reverse PCR primer containing an attB1 site and template specific sequence. Primer sequences are available upon request. PCR products were purified using a Qiagen gel extraction kit (Qiagen, Valencia, California) and were used immediately in BP reactions with pDONR p4-p1R according to the manufacturer's guidelines (Invitrogen Multisite Gateway system, Carlsbad, California). The resulting plasmid was a 5′ entry clone for the Gateway system which was named p5E-8xgli-lc. The p5E-8xgli1-lc entry clone, along with two Multisite Gateway-compatible entry vectors from the Tol2 kit [48]; a middle entry vector carrying the *mCherry RFP* open reading frame named pME-mCherry and a 3′ entry vector carrying a SV40 poly A tail from pCS2+, were incubated in the presence of the LR Clonase II Plus Enzyme mix (Invitrogen) and the destination vector pDestTol2pA2 as previously described [48]. The resulting plasmid contained a Gli-dependent:mCherry reporter construct flanked by the minimal Tol2 transposon elements and was named Gli-d:mcherry reporter plasmid (see Figure 1A). 25–50 pg of reporter plasmid DNA was coninjected along with 30–40 pg of in vitro transcribed Tol2 transposase mRNA into wild type 1–2 cell stage embryos. The strongest expressing mCherry founders were raised to adulthood and outcrossed to wild type embryos. The F1 generation of transgenics was identified by screening for the presence of mCherry expression in progeny. We identified 8 fish that expressed mCherry within regions of known Gli activity in developing embryos. Four of these fish displayed strong expression levels of mCherry and were maintained as our stable lines. Careful examination of the four stable lines failed to detect any distinguishable differences in the pattern of mCherry expression throughout embryonic development.

### mRNA synthesis

Tol2 transposase enzyme mRNA was generated using the pCS2FA-transposase plasmid as a template [48]. Capped mRNA was transcribed from linearized DNA using RNA polymerase in vitro transcription kit, according to the manufacturer's instructions (mMESSAGE, mMACHINE; Applied Biosystems, Foster City, CA).

### Reverse Transcriptase PCR

Total RNA was extracted from groups of 10–20 embryos at stages of development ranging from 0.5 hpf to 30 hpf, using RNA Tissue Extraction and DNase kits (PerfectPure RNA Tissue Kit, 5 Prime). cDNA was synthesized using SuperScript III and random primers (Invitrogen) and PCR was performed using 2× PCR Supermix (Promega, Madison, Wisconsin). Additional control samples, without reverse transcriptase were performed for each sample.

The following primer sequences used were as follows: gli1 forward:

5′CAGACGTCCTCTCGCCTTAC3,′ gli1 reverse: 5′AGTAGCGCTGTCCTTGCATT3,′ disp1 forward: 5′GCTGTAGGGCTTTCTGTGGA3,′ disp1 reverse: 5′GCCACTGTTGGTAGGAGCAT3,′ gapdh forward: 5′GAAGGTGGGAAACTGGTCAT3,′ gapdh reverse: 5′TTGCACCACCCTTAATGTGA3.′ mCherry forward: 5′ CCTGTCCCCTCAGTTCATGT 3,′ mCherry reverse: 5′ CCCATGGTCTTCTTCTGCAT 3,′

### Cyclopamine treatments

Cya (LC Laboratories, Woburn, Massachusetts) was solubilized in 95% ethanol to obtain a stock solution of 50 mM, which was further diluted in zebrafish embryo water to a working stock concentration of 100 µM. Embryos were bathed in the Cya working stock for 4–24 hours at a time. Immediately following treatment, the Cya solution was removed, embryos were washed in multiple rounds of zebrafish embryo water, and then allowed to develop further until 48–96 hpf, wherein they were analyzed by *in situ* hybridization or cartilage staining.

### Cartilage staining

Cartilage analysis was performed as previously described, zebrafish larvae were fixed at 96 hpf and treated with Alcian blue solution dissolved in 80% ethanol/20% glacial acetic (acid alcohol) acid for several hours or overnight. Larvae were de-stained in several washes of acid alcohol before being transferred to a 1% KOH: 3% hydrogen peroxide solution for further clearing of pigment cells. Larval tissue was then digested in trypsin, followed by dissection of PA cartilages which were then flat mounted as previously described [72]. Cartilage preparations were visualized on a Leica MZ16F.

### Whole mount *in situ* hybridization and Fluorescent Imaging

Whole-mount *in situ* hybridization was conducted essentially as previously described [73]. mCherry riboprobe was made by PCR amplifying a 517 basepair fragment spanning the mCherry open reading frame from the pME-mCherry using the following primers: mCherry forward 5′ CCTGTCCCCTCAGTTCATGT 3′ and mCherry reverse 5′ GCATGGACGAGCTGTACAAGTAA 3′ and a Taq polymerase (Promega). PCR product were ran on an agarose gel to confirm the correct basepair size and subsequently purified using the Qiaquick gel extraction kit (Qiagen) and TA-cloned into a pGemTEasy (Promega) vector. Antisense digoxigenin labeled probes were generated against *shh* [47], *gli1* [*15*], *gli2a* [14], *gli2b* [16], *gli3* [*18*] and *sox9a* [58]. Embryos fixed at a stage older than 25 hpf were raised in 2 mM 1-phenyl-2-thiourea in embryo medium to prevent pigmentation. Gene expression patterns were visualized using NBT/BCIP (Roche Ltd, Basel, Switzerland). After staining completed, embryos were cleared in 80% glycerol and visualized on a Leica MZ16F microscope with camera.

Epifluorescence was visualized in live transgenic zebrafish with a Leica MZ16F microscope. For visualizing fluorescent protein in fixed embryos, whole embryos were mounted on slides in 90% glycerol and confocal stacks were generated using a Zeiss Meta 510 laser scanning microscope.

## Supporting Information

Figure S1 Expression of mCherry protein in Tg(Gli-d:mCherry) founder fish. (A–D) Lateral views, anterior to the left, of 24 hpf founder embryos visualized using brightfield (A,B) or fluorescent (C,D) microscopy. mCherry expressing cells could be clearly visualized in the forebrain (arrow in A,B) and myotome (boxed in A,B; see C,D for higher magnification) in some of the embryos injected with the Gli reporter transgene.
(1.18 MB TIF)

Figure S2 Expression of gli genes in craniofacial region during second day of development. Lateral views, anterior to the left, of 30 hpf (A,D,G,J), 36 hpf (B,E,H,K) or 48 hpf (C,F,I,L) stained with riboprobe for gli1 (A–C), gli2a (D–F), gli2b (G–I) or gli3 (J–L).
(5.07 MB TIF)

Figure S3 mCherry protein expression in craniofacial region of double transgenics. Lateral (A,B) or ventral (C) views of confocal stack projections of 48 hpf Tg(Gli-d:mcherry) and Fli1:gfp double transgenic fish. (A) Reporter expression was visible in craniofacial tissues. (B,C) Closer examination within the jaw showed reporter expression in the oe (arrow) and pe (arrowhead) underlying CNC within the second arch. Abbreviations: PA, pharyngeal arch; ov, otic vesicle.
(1.43 MB TIF)

Figure S4 Expression of gli genes in craniofacial region during third day of development. Lateral views, anterior to the left, of 54 hpf (A,C,E,G) or 72 hpf (B,D,F,H) stained with riboprobe for gli1 (A,B), gli2a (C,D), gli2b (E,F) or gli3 (G,H).
(3.11 MB TIF)
