# Supplementary figures and images for: Visualization of Gli Activity in Craniofacial Tissues of Hedgehog-Pathway Reporter Transgenic Zebrafish
Source: PLoS One. 2010 Dec 21;5(12):e14396. doi: 10.1371/journal.pone.0014396 (PMC3006388; doi:10.1371/journal.pone.0014396)

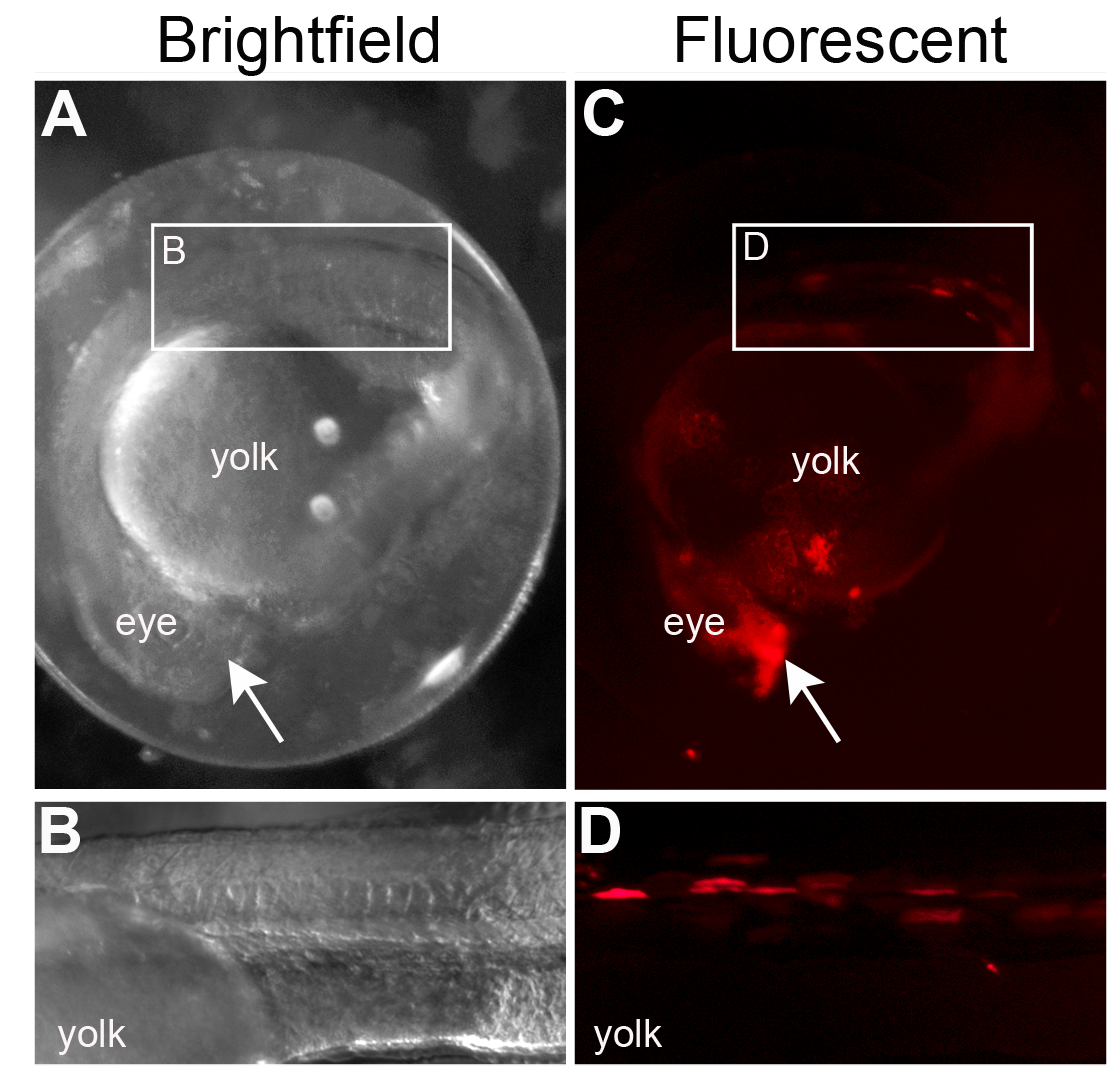

Supplement: Figure S1 — Expression of mCherry protein in Tg(Gli-d:mCherry) founder fish. (A–D) Lateral views, anterior to the left, of 24 hpf founder embryos visualized using brightfield (A,B) or fluorescent (C,D) microscopy. mCherry expressing cells could be clearly visualized in the forebrain (arrow in A,B) and myotome (boxed in A,B; see C,D for higher magnification) in some of the embryos injected with the Gli reporter transgene. (1.18 MB TIF) [file pone.0014396.s001.tif]

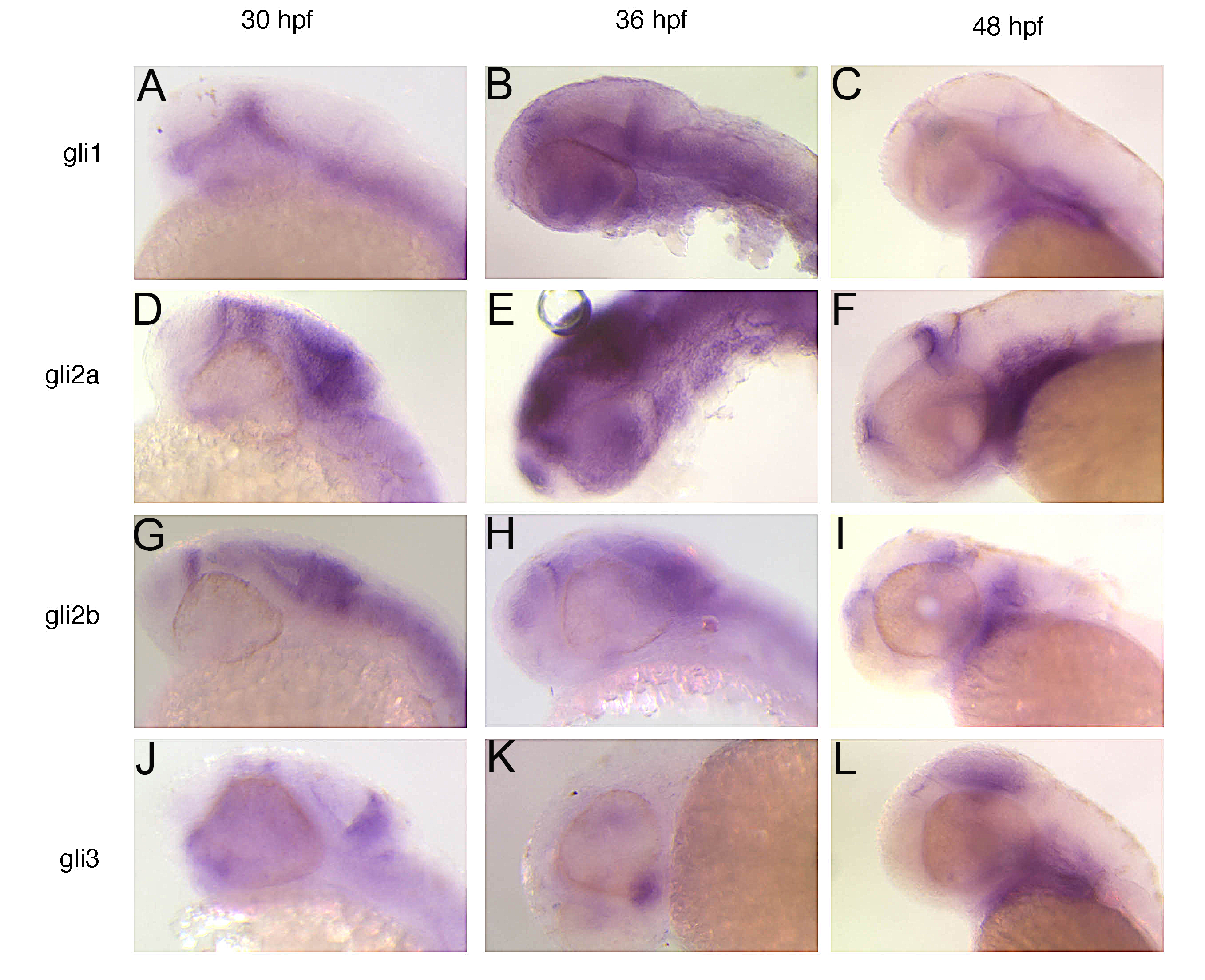

Supplement: Figure S2 — Expression of gli genes in craniofacial region during second day of development. Lateral views, anterior to the left, of 30 hpf (A,D,G,J), 36 hpf (B,E,H,K) or 48 hpf (C,F,I,L) stained with riboprobe for gli1 (A–C), gli2a (D–F), gli2b (G–I) or gli3 (J–L). (5.07 MB TIF) [file pone.0014396.s002.tif]

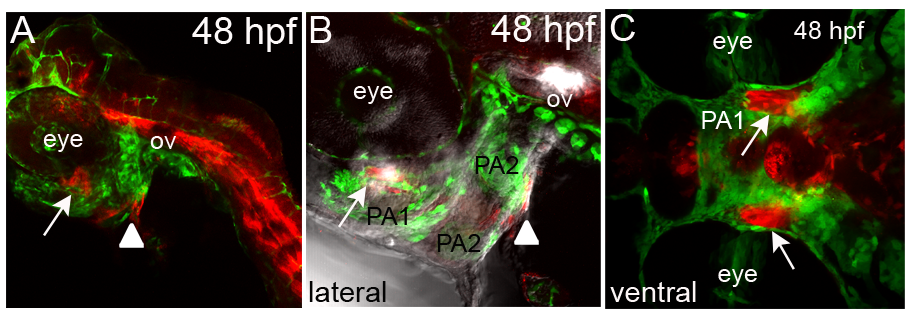

Supplement: Figure S3 — mCherry protein expression in craniofacial region of double transgenics. Lateral (A,B) or ventral (C) views of confocal stack projections of 48 hpf Tg(Gli-d:mcherry) and Fli1:gfp double transgenic fish. (A) Reporter expression was visible in craniofacial tissues. (B,C) Closer examination within the jaw showed reporter expression in the oe (arrow) and pe (arrowhead) underlying CNC within the second arch. Abbreviations: PA, pharyngeal arch; ov, otic vesicle. (1.43 MB TIF) [file pone.0014396.s003.tif]

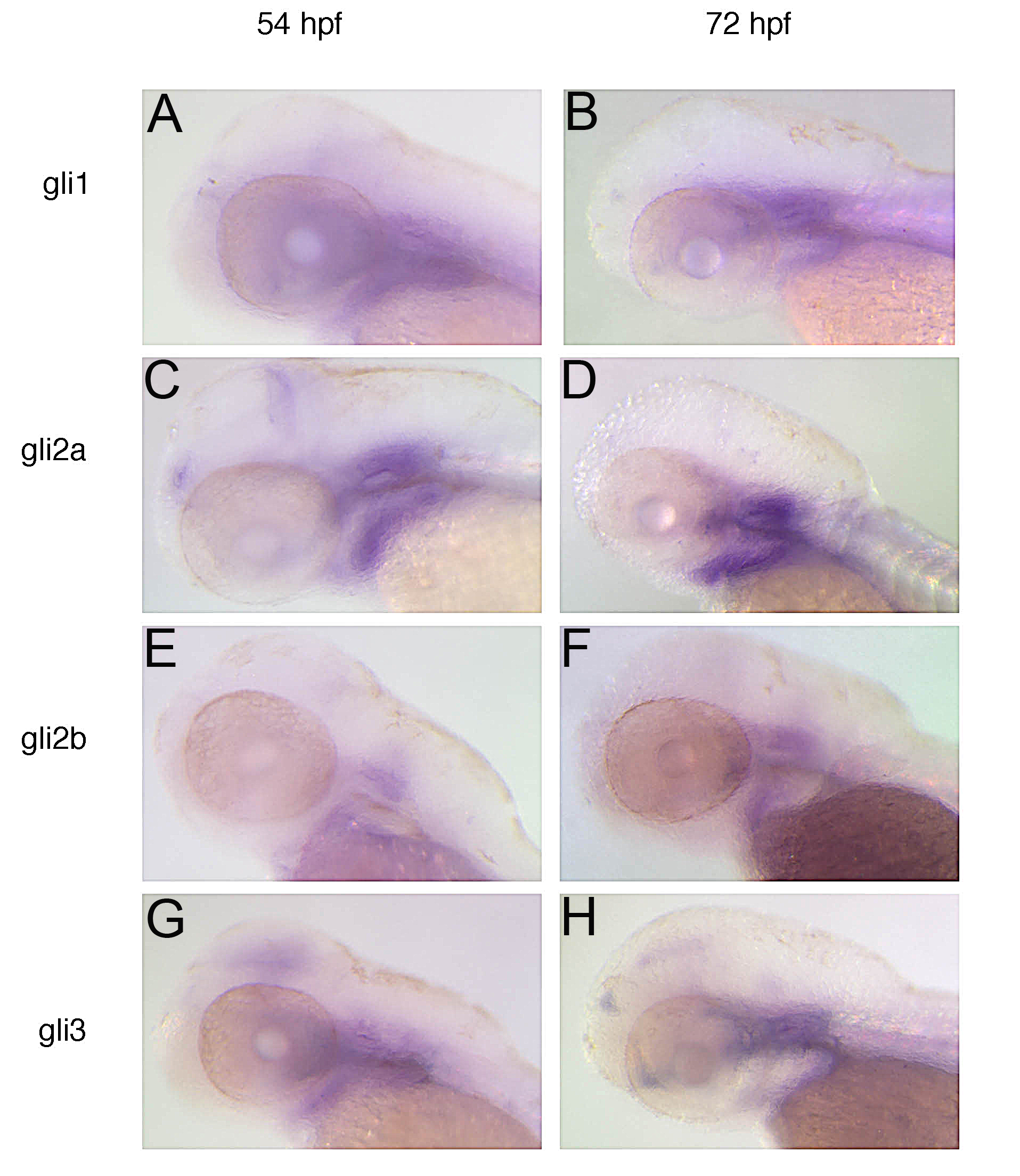

Supplement: Figure S4 — Expression of gli genes in craniofacial region during third day of development. Lateral views, anterior to the left, of 54 hpf (A,C,E,G) or 72 hpf (B,D,F,H) stained with riboprobe for gli1 (A,B), gli2a (C,D), gli2b (E,F) or gli3 (G,H). (3.11 MB TIF) [file pone.0014396.s004.tif]
